# Case Report: 3D Printing Guided Cardiac Autotransplantation for Treatment of a Giant Complex Primary Left Atrial Tumor

**DOI:** 10.3389/fcvm.2021.800459

**Published:** 2021-11-25

**Authors:** Yang-zhao Zhou, Tao Tang, Cheng Luo, Xin-min Zhou, Xian-ming Fu

**Affiliations:** Department of Cardiovascular Surgery, The Second Xiangya Hospital, Central South University, Changsha, China

**Keywords:** cardiac autotransplantation, 3-dimensional printing, primary cardiac tumors, atrial myxoma, surgical resection and reconstruction

## Abstract

Primary cardiac tumors are rare and complete surgical resection is the optimal treatment. However, it is a great challenge to resect some malignant or complex benign left-sided cardiac tumors situated on the posterior aspect of the heart using conventional surgical resection techniques. Previous studies reported that cardiac autotransplantation is a feasible and safe technique for resection of such cardiac tumors. We report a successful case of cardiac autotransplantation with 3-dimensional (3D) printing technique for complete resection of a giant complex primary left atrial tumor.

## Introduction

Most of primary cardiac tumors are benign and can be cured by conventional surgical resection. However, malignant or giant complex primary left cardiac tumors may present a considerable technical challenge to complete resection due to the anatomic limitation and difficult accessibility. The conventional surgical resection may result in local recurrence and poor prognosis. To overcome these technical challenges, a technique of cardiac excision, *ex-vivo* tumor resection with cardiac reconstruction, and cardiac reimplantation termed as cardiac autotransplantation was introduced by Cooley et al. (1) and thereafter has been successfully utilized (2–4). We report a successful case of cardiac autotransplantation using 3D printing for complete resection of a giant complex primary left atrial tumor.

## Case Report

**Figure 1:**
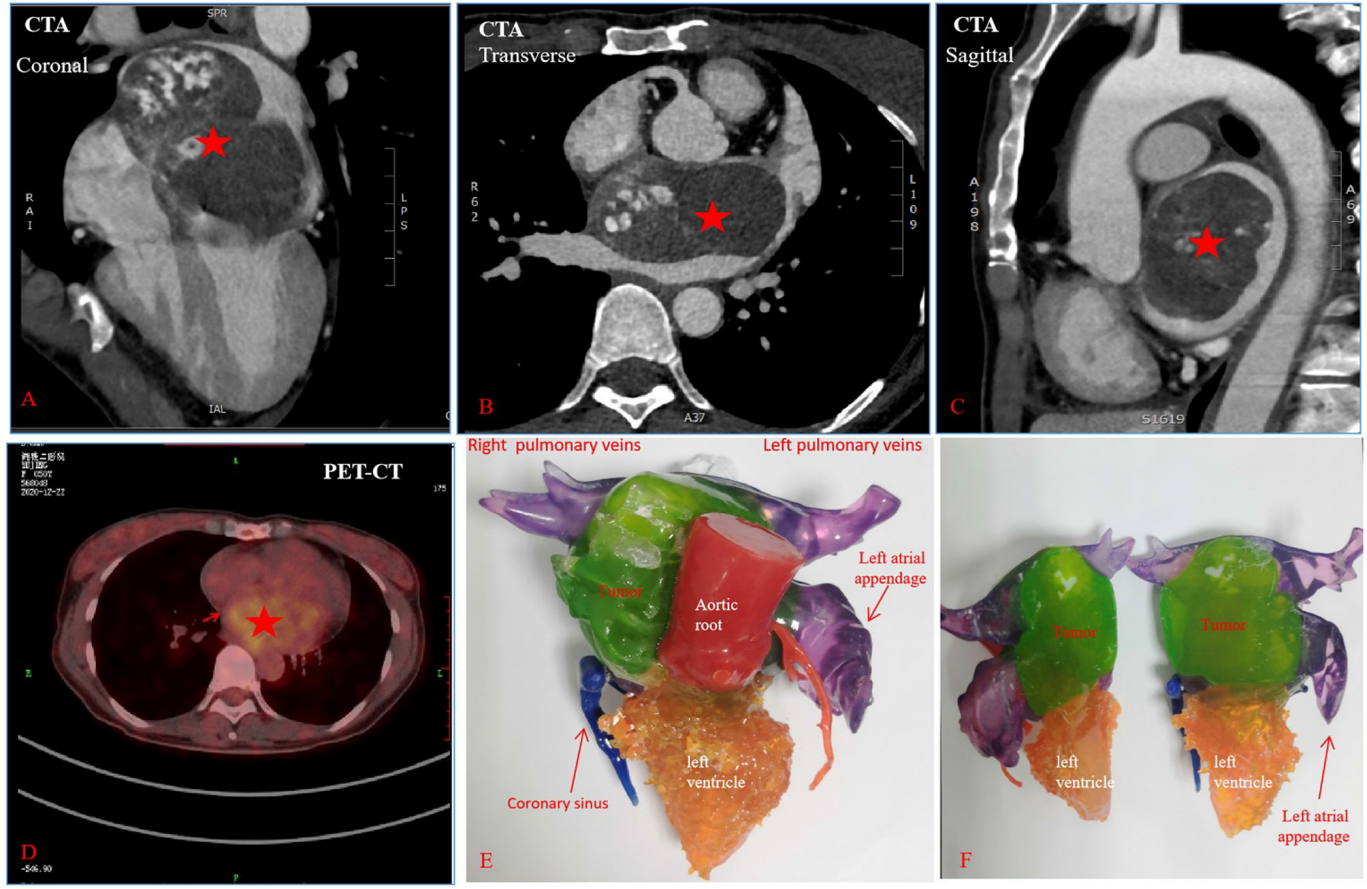
Pre-operative images showing left giant atrial tumor and adjacent structures. **(A–C)** Indicate images from CTA, **(D)** indicates image from PET-CT, **(E,F)** indicate images from 3D printing model. Red asterisks indicate left atrial tumor.

**Figure 2:**
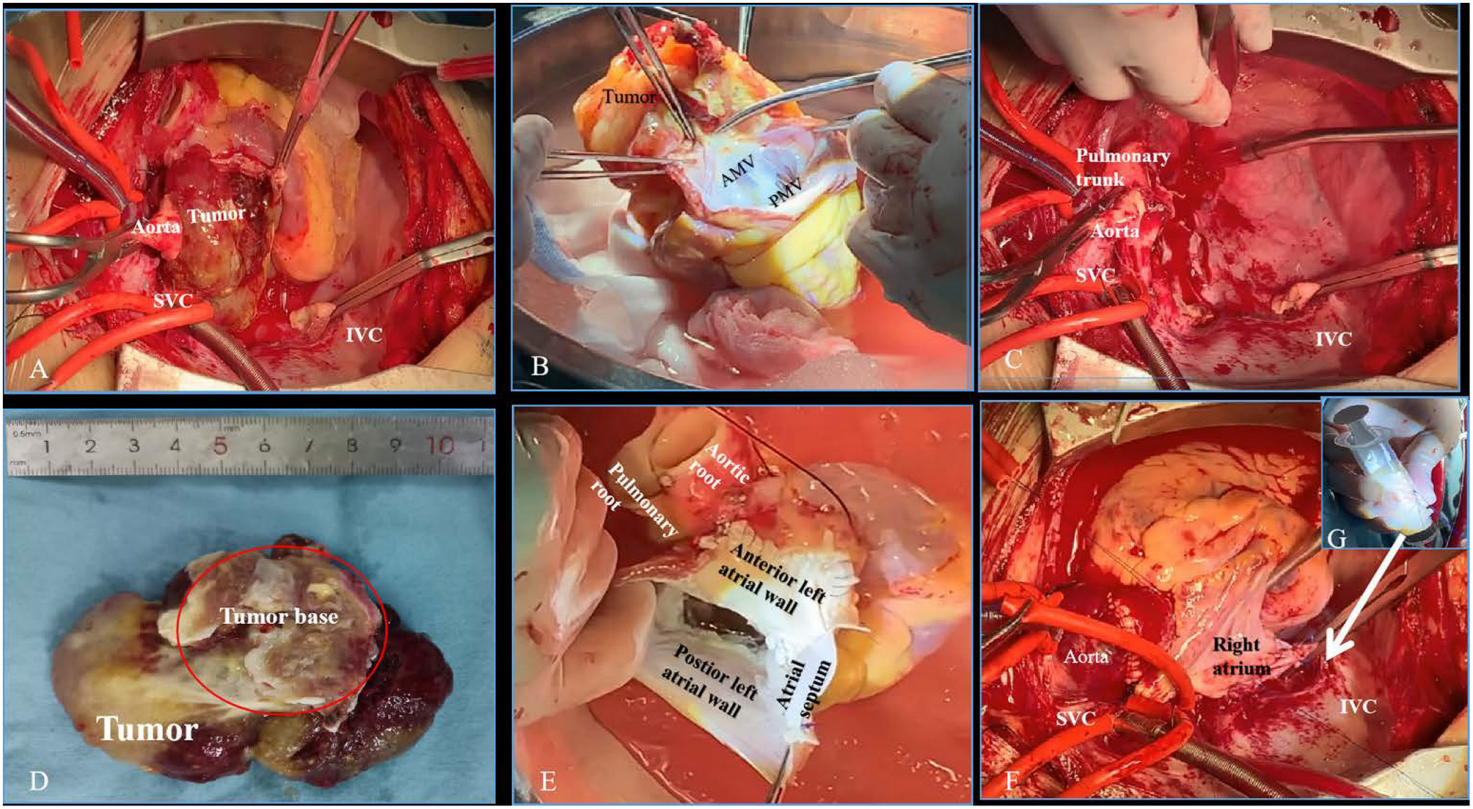
Intraoperative images showing the key surgical steps of cardiac autotransplantation. **(A)** The aorta was divided and the roof of left atrium was then opened to explore the tumor. **(B)** The heart with tumor was excised and the tumor was removed *ex vivo*. **(C)** The pericardial cavity after the resection of the heart. **(D)** The tumor with broad base was completely resected. **(E)** Bovine pericardial patch was used to reconstruct the atrial septum and the anterior wall of left atrium. **(F)** Reimplantation of the heart to the body. **(G)** Self-constructed bovine pericardial tube. SVC, superior vena cava; IVC, inferior vena cava; AMV, anterior mitral valve; PMV, posterior mitral valve.

A 50-year-old previously healthy female presented with dyspnea, fatigue, and 6-kg weight loss during the course of 3 months. She was found to have a giant left atrial tumor by transthoracic echocardiography (TTE). Cardiac computed tomographic angiography (CTA) (Figures 1A–C) showed an 88 × 76 mm large cardiac mass with a broad base involving the atrial septum and the anterior left atrial wall, nearly filled the entire left atrium. The right pulmonary vein orifices and the mitral valve were associated with the tumor. 18F-fluorodeoxy-glucose positron emission tomography-computed tomography (PET-CT) scan demonstrated that left atrial tumor was hypermetabolic tumor activity without signs of any distant metastases (Figure 1D). These preoperative images studies suggested that the left atrial tumor could be malignant. To better understand the adjacent anatomic relationship between left atrial tumor and surrounding tissues, a 3D printing model (Figures 1E,F) was fabricated in collaboration with Changsha Jiebo Information Technology Co., Ltd, China. It was made of photosensitive resin material by 3D printing device (Sailner J501 Series Color Multimaterial 3D printer). The duration of the print run was about 5 h. In view of the extent of the mass and the limited exposure, the conventional surgical resection approach would be difficult and risky. After careful evaluation of the 3D printed model, cardiac autotransplantation was considered to be suitable. Following median sternotomy and full heparinization, the aorta and superior vena cavae and right femoral vein were cannulated, and cavae snared, for total cardiopulmonary bypass. The heart was arrested using antegrade cardioplegic solution (Custodio HTK Solution; Dr Franz Kohler Chemie GMBH, Bensheim, Germany). The heart was excision (Figures 2A–C) and the tumor were removed (Figure 2D). The repair of the excised septum and anterior left atrial wall was performed with two bovine pericardium patches (Figure 2E). Reimplantation of the heart is similar to a bicaval orthotopic cardiac transplant (Figure 2F). Self-constructed bovine pericardial tube has been used as a graft to complete the re-anastomosis of inferior vena cavae to alleviate tension (Figure 2G). Although preoperative images suggested the left atrial tumor was malignant, the final pathology confirmed atrial myxoma. The patient recovered well and was discharged on post-operative day 12. She remains well without recurrence or arrhythmias at 9 months after surgery.

## Comment

Cooley and colleagues reported the first case of cardiac autotransplantation for resection of a cardiac tumor (1). In 2006, Michael J. Reardon and colleagues reported the first series results of cardiac autotransplantation for 11 consecutive patients with malignant or complex primary left-heart tumors (2). There were no operative deaths. Median overall survival was 18.5 months in patients with sarcomas. All patients with benign tumors were alive without evidence of recurrence. In 2014, they extended the series to 34 patients (4). The benign group had no operative deaths and 100% 2-year survival. For primary malignant tumors, survival at 1 and 2 years was 46 and 28%. Surgical outcomes are excellent in patients who do not require concurrent pneumonectomy. Cardiac autotransplantation for resection of complex left-sided tumors have been reported by other heart centers (5–7). These results indicated that cardiac autotransplantation is a feasible and safe technique for resection of complex left-sided tumors. However, the cardiac autotransplantation technique is a major procedure with prolonged cardiac ischemic time. It should be adopted cautiously in experienced centers.

There are few reports on successful uses of 3D printing in planning for cardiac surgery (7, 8). Menegazzo et al. firstly reported on utilization of a 3D printing model to perform cardiac autotransplantation for complete resection of primary left-heart sarcoma and left atrial reconstruction in 2019 (7). 3D printing help surgeons to better understand the spatial anatomic relationship between the tumor and surrounding structures before surgery, which may aid preoperative planning of the operation and selection of the optimal approach.

In summary, cardiac autotransplantation is a well-established cardiac surgical technique. This technique can be taken into consideration for surgical resection of left-sided malignant or complex large benign cardiac tumors. 3D printing technology is valuable to aid in the surgical treatment of complex primary left cardiac tumors with a good outcome.

## Data Availability Statement

The original contributions presented in the study are included in the article/supplementary material, further inquiries can be directed to the corresponding author/s.

## Ethics Statement

The studies involving human participants were reviewed and approved by the Institutional Ethics Committee of the Second Xiangya Hospital of Central South University. The patients/participants provided their written informed consent to participate in this study. Written informed consent was obtained from the individual(s) for the publication of any potentially identifiable images or data included in this article.

## Author Contributions

X-mF and Y-zZ: concept, data interpretation, drafting, critical revision, and approval of article. X-mF, TT, CL, Y-zZ, and X-mZ: critical revision and approval of article. All authors contributed to the article and approved the submitted version.

## Conflict of Interest

The authors declare that the research was conducted in the absence of any commercial or financial relationships that could be construed as a potential conflict of interest.

## Publisher's Note

All claims expressed in this article are solely those of the authors and do not necessarily represent those of their affiliated organizations, or those of the publisher, the editors and the reviewers. Any product that may be evaluated in this article, or claim that may be made by its manufacturer, is not guaranteed or endorsed by the publisher.

## References

[1] Cooley DA Reardon MJ Frazier OH Angelini P. Human cardiac explantation and autotransplantation: application in a patient with a large cardiac pheochromocytoma. Tex Heart Inst J. (1985) 12:171–6. 15227027 PMC341833

[2] Reardon MJ Malaisrie SC Walkes JC Vaporciyan AA Rice DC Smythe WR. Cardiac autotransplantation for primary cardiac tumors. Ann Thorac Surg. (2006) 82:645–50. 10.1016/j.athoracsur.2006.02.086 16863779

[3] Conklin LD Reardon MJ. Autotransplantation of the heart for primary cardiac malignancy: development and surgical technique. Tex Heart Inst J. (2002) 29:105–8; discussion 08. 12075865 PMC116735

[4] Ramlawi B Al-Jabbari O Blau LN Davies MG Bruckner BA Blackmon SH. Autotransplantation for the resection of complex left heart tumors. Ann Thorac Surg. (2014) 98:863–8. 10.1016/j.athoracsur.2014.04.125 25086947

[5] Dolenc J Jelenc M Dimitrovska L Dolenc-Strazar Z Klokocovnik T. Cardiac autotransplantation and extracellular matrix patch reconstruction for a left atrial sarcoma. J Card Surg. (2017) 32:95–6. 10.1111/jocs.13098 28139005

[6] Suzuki K Toda K Saito S Miyagawa S Yoshikawa Y Hata H. Autotransplantation for cardiac sarcoma with fenestrated patch and *in situ* pulmonary vein fixation. Circ J. (2019) 83:1764. 10.1253/circj.CJ-18-0680 30745557

[7] Menegazzo WR Alvarez J Cusimano RJ Geib G Torres FS Clausell N. Modified autotransplant with three-dimensional printing for treatment of primary cardiac sarcoma. J Thorac Cardiovasc Surg. (2019) 157:e41–3. 10.1016/j.jtcvs.2018.08.087 30366749

[8] Schmauss D Haeberle S Hagl C Sodian R. Three-dimensional printing in cardiac surgery and interventional cardiology: a single-centre experience. Eur J Cardiothorac Surg. (2015) 47:1044–52. 10.1093/ejcts/ezu310 25161184

